# Effectiveness of an educational intervention on mealtime support needs for people with dementia in residential care facilities: A cluster-randomized controlled trial

**DOI:** 10.1177/14713012251323658

**Published:** 2025-02-21

**Authors:** Lígia Passos, João Tavares, Melissa Batchelor, Daniela Figueiredo

**Affiliations:** RISE-Health, Department of Education and Psychology, 56062 University of Aveiro, Aveiro, Portugal; Institute of Biomedical Sciences Abel Salazar, University of Porto, Porto, Portugal; RISE-Health, School of Health, 56062 University of Aveiro, Aveiro, Portugal; School of Nursing, 306705 George Washington University, Washington DC, USA; RISE-Health, School of Health, 56062 University of Aveiro, Aveiro, Portugal

**Keywords:** dementia, direct care workers, mealtime support needs, long-term care, educational intervention, cluster randomized controlled trial

## Abstract

**Background:**

People with dementia face numerous challenges during mealtimes, including difficulties with food intake, cutlery use, and maintaining attention. These can lead to severe consequences such as malnutrition and aspiration pneumonia, affecting the well-being of these individuals.

**Aim:**

To determine the effectiveness of an educational intervention in improving mealtime support needs and enhancing the well-being of both individuals with dementia and direct care workers.

**Methods:**

A cluster-randomized controlled trial was conducted in four residential care facilities. The study involved direct care workers and residents with dementia, with facilities randomly assigned to either an intervention or control group. The intervention comprised three weekly 2-hour training sessions, focusing on dementia-related mealtime challenges and practical support strategies. Data were collected at baseline and one-week post-intervention using questionnaires and observational tools to assess caregivers’ skills, burnout levels, and job satisfaction, as well as residents’ mealtime behavior and food intake.

**Results:**

Direct care workers from the intervention group showed significant improvements in knowledge (*p* < .001; d = 0.728) and skills (*p* < .001; d = 0.842) compared to the control group. Additionally, there were notable reductions in burnout levels (*p* = .001; d = 0.466) and higher job satisfaction (*p* = .003; d = 0.410). People with dementia in the intervention group demonstrated better performance at mealtimes.

**Conclusion:**

The educational intervention effectively enhanced direct care workers’ abilities to support people with dementia during mealtimes, leading to better outcomes for both caregivers and residents. Implementing such training programs can improve care quality and alleviate challenges in dementia care.

## Introduction

As global demographics continue to shift towards an aging population, dementia appears as a pressing concern affecting older adults worldwide. The World Health Organization (WHO, 2017) has recognized dementia as a significant contributor to disability and dependency among older adults, affecting an estimated 50 million individuals globally. Projections suggest this number could rise to approximately 152 million by 2050 (ADI, 2022). Portugal had an estimated 200,994 people living with dementia in 2019, a number anticipated to increase to 351,504 by 2050 (Nichols et al., 2022). Although the majority of people with dementia live in the community, 84% globally according to (Wimo et al., 2018), a smaller proportion need to be institutionalized either due to worsening clinical and functional conditions or due to the unavailability of family members for continuous care (Lini et al., 2016). People with dementia living in residential care facilities (RCF) face complex challenges, such as loss of autonomy, social isolation and behavioral changes, requiring specialized care to ensure their well-being (Boumans et al., 2019). Given the rising prevalence of dementia, the condition significantly affects activities of daily living, including mealtime performance, negatively influencing quality of life (Andersen et al., 2004).

Beyond food, calories and nutrients intake, mealtimes provide opportunities for social interaction and the maintenance of meaningful roles, highlighting their importance in preserving quality of life, establishing daily routines, and fostering emotional connections for people with dementia across various care settings (Brush et al., 2002; H. H. Keller et al., 2015). However, people with dementia can experience several challenges during mealtimes, including difficulties such as recognizing food and drink, using cutlery, maintaining attention, following a meal routine, loss of appetite, and dysphagia (Kai et al., 2015). Behavioral changes, like agitation and aggressiveness, and challenging interactions with caregivers can disrupt mealtime experience routines (Porter et al., 2021). Serious health risks, including malnutrition, dehydration, and aspiration pneumonia, are among the critical consequences of mealtime difficulties (Abbott et al., 2013). Additionally, the social and emotional significance of eating and drinking means that mealtime challenges can severely impact mental health and well-being (Burges Watson et al., 2018). Given its psychosocial importance, mealtime challenges can cause distress for both individuals with dementia and their caregivers (Egan et al., 2020; Fetherstonhaugh et al., 2019; H. H. Keller, 2016).

People with dementia may already be dealing with serious weight loss and other mealtime-related issues by the time they enter a RCF, since institutionalization has been associated with the decline of health-related quality of life (de Medeiros et al., 2020). Due to the potential for greater physical and mental decline, care staff may find it difficult to encourage people with dementia to eat and drink properly. Fundamentally, care staff in RCF should have the necessary abilities and expertise to assist those with dementia with eating and drinking (Murphy & Aryal, 2020).

In RCF, direct care workers (DCW) provide hands-on care for older adults, including those living with dementia, and they represent the largest component of the care staff. Previous studies have shown that care staff lacked sufficient nutritional knowledge (Beattie et al., 2014) and were not aware of the special needs and beneficial care practices during mealtimes of people with dementia (Lea et al., 2017). Despite the supporting role DCW play in the daily care of people with dementia, education and training often seem insufficient for DCW to meet the demands of providing high-quality dementia care (Tompkins et al., 2020).

As highlighted in previous literature, educational interventions and training for DCW focusing on mealtime challenges are critical to improving care practices and outcomes for people with dementia (Jung et al., 2024). Studies focused on training for care staff have shown favourable results, such as better knowledge and positive attitudes among DCW, in addition to greater food intake and improved behaviour of people with dementia during meals (Passos et al., 2024b). Faraday et al. (2019) identified a significant need for training interventions that include the importance of person-centred care, strategies, skills and knowledge; and creating the right environment. An educational training-based intervention enhanced with hands-on support for DCW has been shown to be feasible and positive impact on DCW’ knowledge, job satisfaction, and burnout, along with potential improvements in people with dementia’ feeding behaviours (Passos et al., 2024b). Nonetheless, dementia-related behaviours, insufficient training and education in dementia care, a heavy workload, interpersonal conflicts, or unsupportive leadership all lead to high levels of stress, burnout, and job dissatisfaction among DCW, threatening the quality of care and the well-being of the residents (Edberg et al., 2008; Gray-Stanley & Muramatsu, 2011). Addressing these challenges through target educational interventions could enhance DCW’ skills and reduce their stress and directly impact the quality of mealtime care for people with dementia (Li et al., 2021; Reimer & Keller, 2009).

The research question guiding this study was: does an educational intervention improve the skills, reduce burnout, and increase job satisfaction of DCW, as well as enhance mealtime experiences for people with dementia living in RCF? To address this, the study aims to analyze the effects of an educational intervention designed to improve mealtime care practices by enhancing DCW skills in providing person-centred assistance during meals. Ultimately, the intervention seeks to improve both the quality of feeding assistance and the mealtime experiences of residents with dementia.

Through rigorous assessment, this research aims to contribute to the development of evidence-based interventions tailored to optimize the care for individuals living with dementia in residential care facilities.

## Methods

### Design and settings

This study was designed as a 5-week cluster-randomized controlled trial (RCT), with an educational intervention, conducted in four non-profit RCF from urban regions in the north and center of Portugal, between September and December 2023. The facilities did not belong to the same organizations; however, they are all Private Non-profit Institutions of Social Solidarity (*IPSS – Instituições Particulares de Solidariedade Social*), that operate in conjunction with the National Social Security. Table 1 shows some characteristics of the RCS where the study was conducted.

**Table 1.** Characteristics of the residential care facilities.

	Intervention group		Control group	
Residential care facility	1	2	3	4
% of residents with dementia	51.6	67	54	52
Ratio residents x DCW per work shift	10.3	10	10.3	12
Ratio residents with dementia x DCW per work shift	5.3	6.6	5.6	6.3

DCW: direct care workers.

Recognizing the possibility of contamination between the intervention and control groups within the same care setting, particularly in a context where participants may regularly interact with each other, cluster randomization was chosen to mitigate this risk, ensuring that each RCF be assigned exclusively to an intervention or control group. Therefore, RCF were grouped into clusters based on the ratio of morning shift staff to residents with dementia (5:1 or 6:1) to ensure comparability between facilities. Following clustering, randomization was performed using an online research randomizer tool (https://www.randomizer.org/), which assigned facilities to either the intervention group (IG) or the control group (CG). This process ensured that group allocation was unbiased and adhered to rigorous randomization principles. This study adhered to the CONSORT guidelines for reporting the results of the randomized trial (Appendix 1).

This study was approved by the Ethics Committee of the Health Sciences Research Unit-UICISA: E (Ref. 837/01-2022). Written consent forms were obtained previous to data collection and study participation according to the Declaration of Helsinki. Confidentiality was guaranteed by assigning each participant a numerical code. People with dementia who are unable to express themselves verbally or in writing have undergone an assent process. The primary researcher provided residents with dementia with straightforward information, using simple language, visual aids, and short sentences to enhance comprehension about the study before the assessment. Before the meal observation, participants were asked for their permission. Their assent was presumed if they showed no evidence of objection or distress. This assent process was based on previous protocol (Batchelor-Aselage et al., 2014) and the pilot study of this intervention (Passos et al., 2024b).

### Participants

This study involved two groups of participants: DCW and residents with dementia. The educational intervention was exclusively designed for DCW, who were given flexibility in selecting the most convenient session times. Residents with dementia were also included as participants to evaluate the intervention’s impact on mealtime performance, as they are supported by the trained DCW.

The primary group of participants included DCW aged 18 years or older, employed at the RCF for a minimum of 3 months. Trainees were not included in the study.

Residents aged 65 years or older, with a clinical diagnosis of dementia recorded in the individual data registration file, who lived in the RCF for at least two months, required assistance during meals, and had a legal proxy to sign the consent form were also part of the study. Residents receiving tube feeding were not included.

The recruitment process was led by the principal researcher, with support from the RCF technical director, who identified potential participants based on the specified criteria and enabled communication with the residents’ proxies.

### Intervention

The design of the FOoD-EAT (Feeding Older adults with Dementia – Eat, Assist and Train) was established by models and specialized training addressing the mealtime support needs of people with dementia (Amella & Batchelor-Aselage, 2014; Batchelor, 2020). Additionally, insights from a scoping review conducted by the research team (Passos et al., 2024a) and previous interviews with DCW were incorporated to understand their needs concerning mealtime support for people with dementia. The intervention was previously tested in a pilot pre-post intervention study that demonstrated it to be feasible and acceptable for DCW (Passos et al., 2024b).

The intervention was conducted in a group format at the workplace during working hours, with session times previously established by the RCF technical director to avoid disrupting assistance to residents. Two dates were scheduled per session, allowing DCW to choose the most convenient option for them.

The aim of the intervention was to offer knowledge regarding dementia and the mealtime support needs of people with dementia, while also equipping DCW with evidence-based approaches to effectively manage these mealtime support needs. The intervention group received an educational intervention consisting of 3 weekly sessions of 2 hours, totaling 6 hours. Each of the three sessions had educative and practical components (Table 2) and was conducted by a speech therapist with educational training in gerontology (the trainer). An empathetic approach was applied, encouraging active participation, sharing of experiences, and clarification of doubts from the participants. Individual “in-service” support was provided during mealtimes in the two days that followed each training session. The trainer remained present within the RCF to assist DCW with the strategies they had learned during practical sessions and to reinforce the educational content of the intervention.

**Table 2.** FOoD-EAT sessions’ outline.

Session	Theme	Educational component	Practical component
1	Dementia	Theoretical information regarding dementia including warning signals, types and stages, risk factors, and cognitive, functional, and sensory symptoms	Simulations of sensory impairment like diminished touch, taste, vision, and fine motor abilities
	Feeding and mealtime	Factors that determine nutritional status, as well as the effects of inadequate nutrition. The importance of mealtimes for health, culture, and socialization. Mealtime challenges, oral *versus* tube feeding	Role-plays to identify signs of mealtime support needs
2	C3P model	Dimensions involved at mealtime: Person with dementia, caregiver and environment	Do’s and don’ts: direct care workers’ techniques and interactions with individuals with dementia
	Strategies	Successful communication with people with dementia and mealtime support strategies, including environment modifications (such as lighting and noise adequation)	Handfeeding techniques (over-hand, under-hand and direct hand)
3	Conclusion	Content recap and integration; evaluation of the intervention program	N/A

C3P: Change the person, change the caregiver, change the place; N/A not applicable

### Data collection and outcome measures

Data were collected one week before the intervention (baseline) and one week after (T1). For the control group, data collection occurred at 4-week intervals.

DCW completed an anonymous sociodemographic questionnaire covering age, sex, education, and other variables. They were asked if they had witnessed any behavior or incidents while assisting a person with dementia to eat and drink and about their self-perceived knowledge and preparedness to assist people with dementia. The questionnaire included Likert scale questions on feelings and attitudes when assisting a person with dementia to eat and drink, self-confidence, and expectations regarding the active participation of people with dementia in meals. The sociodemographic questionnaire for people with dementia included age, sex, education, marital status, use of assistive devices, and food consistency.

The impact of the intervention was assessed through standardized measures to determine DCW burnout levels, job satisfaction, and skills to feed people with dementia. Burnout was evaluated using the Portuguese version of the Maslach Burnout Inventory (MBI) (Melo et al., 1999) , which consists of 22 items divided into three subscales: emotional exhaustion (EE), depersonalization (DP), and personal accomplishment (PA). Participants responded on a 7-point Likert scale ranging from 0 = never to 6 = every day. EE measures feelings of being overwhelmed, DP assesses attitudes towards individuals receiving care, and PA evaluates feelings of incompetence and lack of success at work. Higher scores on EE and DP subscales indicate higher levels of burnout, while lower scores on the PA subscale suggest burnout. The internal consistency of the Portuguese version of MBI was satisfactory, with a Cronbach’s alpha of 0.75 for the overall scale and reliability coefficients of 0.80, 0.71, and 0.70 for the EE, DP, and PA subscales, respectively (Melo et al., 1999).

Job satisfaction among DCW was assessed using the Portuguese version of the Minnesota Satisfaction Questionnaire (MSQ) – short form (Ferreira et al., 2009), that includes 20 items rated on a 5-point Likert-type scale, ranging from 1 = extremely dissatisfied to 5 = extremely satisfied. Lower total scores indicate lower levels of job satisfaction. The MSQ short form can assess two dimensions of job satisfaction: intrinsic and extrinsic. Intrinsic satisfaction concerns to feelings about the work itself, while extrinsic satisfaction relates to external aspects of the work situation. The Portuguese version of MSQ – short form demonstrated excellent internal consistency (α = 0.93) (Ferreira et al., 2009).

The skills of DCW in assisting people with dementia during mealtimes were assessed using the Portuguese version of the Feeding Skills Checklist (FSC) (Passos et al., 2021a). The FSC comprises 24 items related to feeding practices, categorized into three dimensions: person with dementia, caregiver, and environment. The person with dementia dimension evaluates food preferences, meal routines, and conditions to receive a meal. The caregiver dimension assesses caregiver skills and interactions with the person with dementia during meals, while the environment dimension evaluates dining room conditions to improve the dining experience for people with dementia. During meal observations, the items are evaluated for presence, absence, or inapplicability to the condition, with one point assigned for each practiced action. A higher final score indicates greater overall skills, and the scores can also be analysed by dimensions to identify the specific areas where the caregiver has more, or less, ability to provide care. The Portuguese version of the FSC demonstrated very satisfactory inter-observer reliability (κ = 0.844) (Passos et al., 2021a). The FSC was completed by the main researcher during lunch (the most complete meal of the day, consisting of soup, main course, and dessert) to a person with dementia participating in the study, on two consecutive days or not, depending on the DCW’ work schedule. The mean of the final score obtained in the two assessments was considered for data analysis.

Finally, it was also applied a knowledge test, developed by the study´s authors, comprising 10 multiple-choice questions concerning dementia and mealtime support needs. This test was pilot-tested with 3 potential respondents to refine its intelligibility, adequacy, and relevance of the questions. The knowledge test was also used in the pilot study of the intervention (Passos et al., 2024b). Participants completed the test prior to the initial training session and again after the final session. As the control group did not receive the training sessions, they only performed the test at baseline.

For people with dementia, outcome measures comprised time spent on a meal, determined by activating a stopwatch at the meal’s start and stopping it at the end of ingestion; food intake, assessed through visual percentage estimation of one meal’s intake (Toledo et al., 2018); and the need for assistance and feeding difficulties, evaluated using the Portuguese version of the Edinburgh Feeding Evaluation in Dementia (EdFED) (Passos et al., 2021b). EdFED is a questionnaire consisting of 10 items developed to assess the need for assistance during meals, challenges correlated with eating, and patterns of feeding behaviours. Scores range from 0 to 20, with higher scores indicating greater modifications in feeding behaviours. The Portuguese version of EdFED demonstrated reasonable internal consistency (Cronbach’s α = 0.705) and highly satisfactory interobserver reliability (Cohen’s κ = 0.882) (Passos et al., 2021b). These data were collected during the observation of two lunches offered by DCW participating in this study.

Table 3 summarises the outcome measures and corresponding assessment instruments used in this study.

**Table 3.** Summary of the outcome measures of the study.

Level of assessment	Variables	Assessment instruments	
		Direct care workers	People with dementia
Sample characterization	Sociodemographic data	Anonymous questionnaire^a^	Anonymous questionnaire^b^
	Knowledge and preparedness	5 points Likert-scale questions^a^	-
	Feelings and attitudes	10 points Likert-scale questions^a^	-
	Self-confidence	10 points Likert-scale questions^a^	-
	Expectations	5 points Likert-scale questions^a^	-
Impact of the intervention	Burnout	Maslach burnout Inventory^a^	-
	Job satisfaction	Minnesota satisfaction Questionnaire^a^	-
	Feeding skills	Feeding skills Checklist^c^	-
	Knowledge about dementia	Knowledge test^a^	-
	Time spent on a meal	-	Stopwatch^c^
	Food intake	-	Visual estimation of intake percentage^c^
	Feeding difficulties/support needs	-	Edinburgh Feeding Evaluation in Dementia^c^

^a^Self-report assessment.

^b^Information from individual data registration file.

^c^Observation-based assessment.

### Data analysis

Descriptive statistics were calculated for sociodemographic characteristics of participants at baseline. Quantitative data has a normal distribution, assessed using the Shapiro-Wilk test. Therefore, they are presented as mean and standard deviation, and the differences were analyzed by the Student’s t-test. Nominal variables were compared using the Chi-Square test with Bonferroni correction to identify categories that differ between the two groups. The results of the comparison of data between groups at baseline and T1 with non-normal distribution shown by the Shapiro-Wilk test are presented as median and quartiles.

For the analysis of baseline and T1 simultaneously, the generalized estimation equation (GEE) analysis was applied to the comparison between groups, which allows this simultaneous analysis even in non-normal data. Effect sizes were calculated (Cohen´s D) and interpreted as 0.2 - < 0.5 (small effect), 0.5 - < 0.8 (moderate effect) and >= 0.8 (large effect)(Cohen, 1988). All statistical analyses were performed using IBM SPSS Statistics (Version 25), and a *p*-value <.05 was considered significant.

## Results

**Figure 1.**
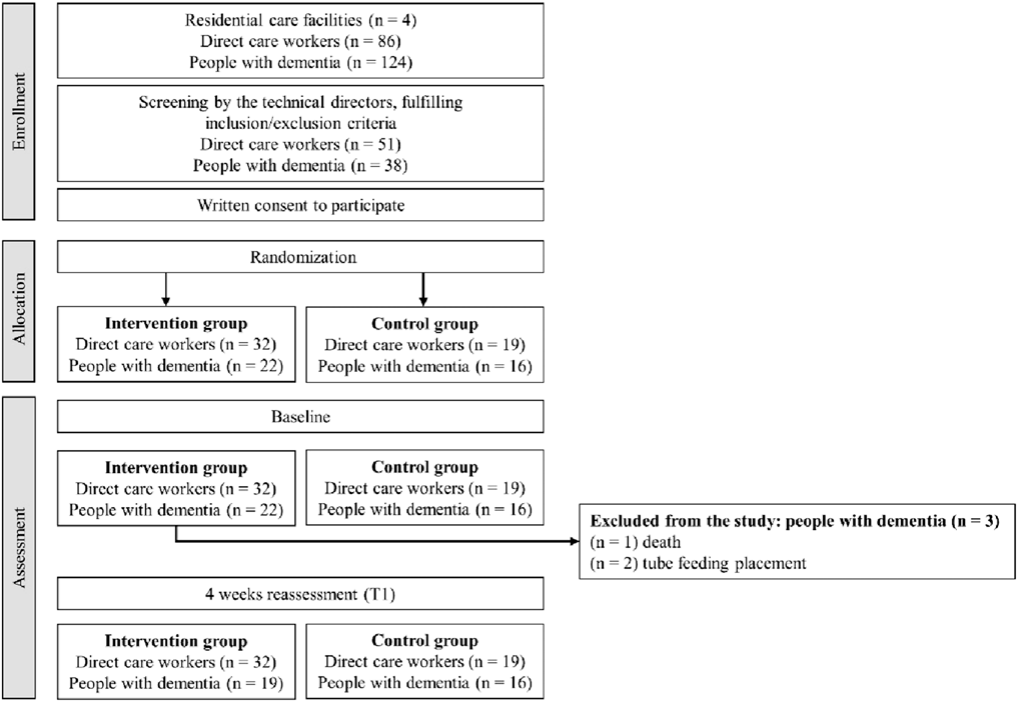
Flowchart showing enrollment to the study.

A flowchart of the enrolment, allocation and data collection is presented in Figure 1.

### Participant characteristics

The majority of participants were female (98%) with a median age of 50 (39-56) years old. Of the participants, 43.2% were married, and 47% had completed high school (12 years of education). Participants have worked as DCW for 5 years and have been employed by the RCF for 4 years. Most of participants (78.4%) reported never having received specialized training on mealtime support needs of people with dementia. Sociodemographic data did not show any significant differences between the groups of DCW (Table 4).

**Table 4.** Baseline characteristics of direct care workers.

	Total (*n* = 51) N (%)	Intervention group (*n* = 32) N (%)	Control group (*n* = 19) N (%)	*p*	*d*
Sex					
Female	50 (98)	32 (100)	18 (94.7)	0.373	0.185
Male	1 (2)	0 (0)	1 (5.3)		
Marital status					
Unmarried	29 (56.8)	17 (53.1)	12 (63.2)	0.484	0.108
Married	22 (43.2)	15 (46.9)	7 (36.8)		
Education					
4 years	8 (15.6)	5 (15.6)	3 (15.8)	0.836	0.168
5–9 years	18 (35.2)	10 (31.2)	8 (42.1)		
10–12 years	24 (47)	16 (50)	8 (42.1)		
College degree	1 (2.2)	1 (3.2)	0 (0)		
Training					
No	40 (78.4)	25 (78.1)	15 (78.9)	0.999	0.010
Yes	11 (21.6)	7 (21.9)	4 (21.1)		
Other sociodemographic variables - median (Q1-Q3)					
Age [years]	50 (39–56)	51 (39–56)	49 (37–57)	0.891	0.019
Time profession [years]	5 (3–11)	5 (2.3–14.8)	4.5 (3–10)	0.646	0.064
Time in the RCF [years]	4 (2–9)	4 (2–11)	3 (1–9)	0.324	0.138

RCF: residential care facility; *p* = *p* value; *d* = Cohen’s d effect size.

Thirty-five people with dementia participated in the study. The majority were female (80%), with a mean age of 84.6 years (7.33). Most of participants were widowed (71.5%) and have 4 years of education (74.2%). Only 2 participants (5.7%) were bedridden. Most participants (62.8%) did not use any type of prosthesis and received pureed consistency meals (60%). There are differences between the control and experimental groups in terms of education, use of assistive devices and food consistency (Table 5).

**Table 5.** Baseline characteristics of people with dementia.

	Total (*n* = 35) N (%)	Intervention group (*n* = 19) N (%)	Control group (*n* = 16) N (%)	*p*	*d*
Sex					
Female	28 (80)	14 (73.7)	14 (87.5)	0.415	0.172
Male	7 (20)	5 (26.3)	2 (12.5)		
Marital status					
Single	3 (8.5)	2 (10.5)	1 (6.2)	0.489	0.263
Married	4 (11.5)	1 (5.3)	3 (18.8)		
Divorced	3 (8.5)	1 (5.3)	2 (12.5)		
Widowed	25 (71.5)	15 (78.9)	10 (62.5)		
Education					
No education	4 (11.4)	4 (21.1)	0 (0)	**0.024**	0.519
4 years	26 (74.2)	15 (78.9)	11 (68.7)		
10–12 years	1 (3)	0 (0)	1 (6.3)		
College degree	4 (11.4)	0 (0)	4 (25)		
Bedridden					
Yes	2 (5.7)	2 (10.5)	0 (0)	0.489	0.226
No	33 (94.3)	17 (89.5)	16 (100)		
Assistive devices					
None	22 (62.8)	17 (89.5)	5 (31.3)	**0.003**	0.634
Eyeglasses	6 (17.1)	2 (10.5)	4 (25)		
Dental	2 (5.9)	0 (0)	2 (12.5)		
Eyeglasses and dental	5 (14.2)	0 (0)	5 (31.3)		
Food consistency					
Regular	12 (34.2)	4 (21.1)	8 (50)	**0.030**	0.447
Soft	2 (5.8)	0 (0)	2 (12.5)		
Pureed	21 (60)	15 (78.9)	6 (37.5)		
**Age** [years] mean (SD)	84.6 (7.33)	84 (1.7)	85 (1.9)	0.471	0.552

SD: standard deviation; *p* = *p* value; *d* = Cohen’s d effect size.

DCW were asked at baseline whether they had witnessed any behaviors or incidents while assisting a person with dementia to eat and drink (Table 6). There are significant differences in the observation of agitation (*p* < .001) and sleepiness (<0.001) which was higher in the control group, and aggression (*p* = .012), higher in the experimental group.

**Table 6.** Behaviors and incidents witnessed during mealtime assistance.

	Intervention Group (*n* = 32)	Control Group (*n* = 19)	*p*	*d*
Agitation	7 (21.9%)	16 (84.2%)	**<0.001**	0.635
Sleepiness	7 (21.9%)	18 (94.7%)	**<0.001**	0.705
Aggression	29 (90.6%)	11 (57.9%)	**0.012**	0.385
Refuse to eat	30 (93.8%)	19 (100%)	0.523	0.156
Retain food in the mouth	29 (90.6%)	17 (89.5%)	0.999	0.019
Spit out food	31 (96.9%)	16 (84.2%)	0.14	0.228
Choking	29 (90.6%)	18 (94.7%)	0.999	0.074
Clench teeth	31 (96.9%)	18 (94.7%)	0.999	0.053

*p* = *p* value; *d* = Cohen’s d effect size.

Table 7 shows the results for questions related to feelings, self-confidence, expectations and knowledge/preparation reported by DCW. At baseline, the IG had a higher level of difficulty to assist people with dementia to eat and drink than the CG (*p* = .043), but there was a significant reduction after the intervention (*p* = .001), while the CG had a significant increase (*p* = .039). There were no significant differences between the groups at T1. Both groups had significant increases in gratification levels during the study (*p* = .016 for CG and *p* < .001 for IG), with the IG having a more significant increase in the re-assessment (*p* = .004). The CG showed a significant increase (*p* = .007) in physical overload, while the IG showed a reduction (*p* = .011), but there were no significant differences between the groups in any phase of the study. The IG showed a significant reduction in emotional overload (*p* = .001), and there was a significant difference between the groups with this reduction in IG in the second assessment (*p* = .013).

**Table 7.** Feelings, self-confidence, expectations and knowledge/preparation of direct care workers.

	Intervention group (*n* = 32)			Control group (*n* = 19)				
	T0 Median (Q1 – Q3)	T1 Median (Q1 – Q3)	Within-group difference *p* (*d*)	T0 Median (Q1 – Q3)	T1 Median (Q1 – Q3)	Within-group difference *p* (*d*)	Between-group difference (T0) *p* (*d*)	Between-group difference (T1) *p* (*d*)
Levels while feeding a PWD								
Difficulty	5.5 (3–8.8)	5 (3–7)	**0.001** (0.546)	3 (1–6)	5 (2–6)	**0.039** (0.472)	**0.043** (0.283)	0.381 (0.123)
Gratification	9 (7–10)	10 (10–10)	**<0.001** (0.696)	9 (8–10)	10 (8–10)	**0.016** (0.554)	0.531 (0.088)	**0.004** (0.406)
Physical overload	5.5 (3–8)	5 (4–7)	**0.011** (0.499)	5 (1–7)	5 (4–7)	**0.007** (0.619)	0.157 (0.198)	0.628 (0.068)
Emotional overload	7 (5–9)	5 (5–7)	**0.001** (0.600)	7 (4–10)	7 (5–8)	0.168 (0.316)	0.992 (0.0019	**0.013** (0.348)
Self-confidence								
Make PWD actively participate	7.5 (6.3–9)	10 (9.3–10)	**<0.001** (0.792)	9 (6–10)	9 (6–10)	0.180 (0.308)	0.279 (0.152)	**0.009** (0.364)
Feed a dependent PWD	8 (7–9)	10 (9–10)	**<0.001** (0.779)	9 (8–10)	8 (7–9)	0.047 (0.455)	0.200 (0.180)	**<0.001** (0.534)
Assist agitated/aggressive PWD	7 (4.3–9)	9 (9–9)	**<0.001** (0.761)	8 (6–9)	8 (6–9)	0.414 (0.187)	0.181 (0.188)	**0.012** (0.351)
Feed PWD who refuse to eat	7.5 (4.3–9)	9 (9–10)	**<0.001** (0.738)	8 (6–9)	8 (6–9)	0.317 (0.229)	0.425 (0.112)	**0.003** (0.419)
Interact/communicate with PWD	8 (6–10)	10 (9.3–10)	**<0.001** (0.697)	9 (8–10)	10 (9–10)	0.492 (0.158)	0.150 (0.202)	**0.048** (0.276)
Expectations								
Maintain ability to eat for longer	1 (1–2)	1 (1–1)	**0.003** (0.530)	1 (1–3)	1 (1–3)	0.414 (0.187)	0.423 (0.112)	**<0.001** (0.553)
Easier and more rewarding work	1 (1–2)	1 (1–1)	**0.004** (0.493)	1 (1–3)	1 (1–3)	0.157 (0.324)	0.284 (0.150)	**<0.001** (0.512)
Increase job satisfaction	1 (1–1.8)	1 (1–1)	**0.009** (0.459)	1 (1–3)	1 (1–3)	0.102 (0.375)	0.378 (0.123)	**0.001** (0.469)
Developing an important care	1 (1–1)	1 (1–1)	**0.016** (0.427)	1 (1–2)	1 (1–3)	0.194 (0.298)	0.373 (0.125)	**0.001** (0.469)
Feel proud of the work	1 (1–1.8)	1 (1–1)	**0.010** (0.453)	1 (1–1)	1 (1–3)	0.317 (0.229)	0.999 (0.000)	**0.002** (0.424)
**Knowledge/Preparation**	2 (1–2)	2 (1–3)	**<0.001** (0.618)	2 (1–2)	2 (1–2)	0.999 (0.000)	0.585 (0.076)	**0.023** (0.318)

T0 = baseline assessment; T1 = second assessment: *p* = *p* value; *d* = Cohen’s d effect size; PWD = person with dementia.

At baseline, the CG demonstrated higher median scores in self-confidence items compared to the IG (e.g., CG median = 9; IG median = 7.5), suggesting a greater level of self-confidence prior to the intervention. However, there was a significant increase in all items of the IG self-confidence assessment, and also a significant difference between the groups at T1.

Significant changes in the expectations of the benefits of encouraging active participation of people with dementia at mealtimes were observed only in the IG. These expectations included maintaining residents’ ability to eat for longer, making care work easier and more rewarding, increasing job satisfaction, developing meaningful care, and fostering a sense of pride in their work. However, in a positive way, only the expectation that it will increase job satisfaction improved significantly in the IG (*p* = .009), and with a significant difference between the groups at T1 (*p* = .001).

Self-perceived knowledge/preparation to assist people with dementia at mealtimes increased significantly in IG (*p* < .001). There was a significant difference between the groups at T1 (*p* = .023).

### Outcome measures

Regarding burnout, in the emotional exhaustion subscale, the CG showed a significant increase (*p* = .001) while the IG showed a significant reduction (*p* < .001). There was a significant difference in the comparison between the groups at T1 (*p* = .001). Both groups showed a significant reduction in the depersonalization subscale (*p* = .017 in the CG and *p* = .000 in the IG), but there was no significant difference between the groups. Only the IG showed an increase in burnout levels in the personal accomplishment subscale, with a significant reduction in the score (*p* < .001).

Regarding job satisfaction, IG showed a positive change from baseline to T1 since the total MSQ total score increased meaningfully (*p* < .001) while reduced significantly in CG (*p* = .004), with a noteworthy difference between the groups at T1 (*p* = .003). IG showed higher levels of job satisfaction in the intrinsic dimension (*p* < .001). There was a significant difference between the groups at T1 (*p* = .003). In the extrinsic dimension, both groups showed a reduction in job satisfaction, but this was significant only in CG (*p* = <0.001).

The IG showed a positive significant increase, in all FSC dimensions, as well as in the total score: person with dementia dimension (*p* = .007), caregiver dimension (*p* < .001), environment dimension (*p* = .012), total score (*p* < .001). There was a significant difference between the groups at T1 in the caregiver (*p* < .001) and environment (*p* < .001) dimensions.

The IG showed significant improvement in the knowledge test (*p* < .001). The groups did not show differences at baseline, and it was not possible to evaluate differences in T1 since the CG only did the test in the initial assessment. Table 8 shows the outcomes of DCW.

**Table 8.** Direct care workers’ outcomes.

	Intervention group (*n* = 32)			Control group (*n* = 19)				
	T0 Median (Q1 – Q3)	T1 Median (Q1 – Q3)	Within-group difference *p* (*d*)	T0 Median (Q1 – Q3)	T1 Median (Q1 – Q3)	Within-group difference *p* (*d*)	Between-group difference (T0) *p* (*d*)	Between-group difference (T1) *p* (*d*)
Burnout								
Emotional exhaustion	15.5 (12–26)	12 (8.5 – 18.8)	**<0.001** (0.829)	19 (12–25)	22 (16–26)	**0.001** (0.783)	0.815 (0.033)	**0.001** (0.466)
Depersonalization	4.5 (0–7.8)	1 (0–3)	**<0.001** (0.678)	2 (0–6)	1 (0–3)	**0.017** (0.546)	0.340 (0.133)	0.751 (0.044)
Personal accomplishment	7 (3–14)	4.5 (2–7)	**<0.001** (0.790)	8 (6–13)	8 (3–12)	0.192 (0.299)	0.689 (0.056)	**0.010** (0.363)
Job satisfaction								
Intrinsic	48 (42–51.8)	50.5 (46.5–53)	**<0.001** (0.819)	46 (44–50)	45 (44–50)	0.164 (0.319)	0.591 (0.075)	**0.001** (0.452)
Extrinsic	20.5 (17–23.8)	19.5 (17.3 – 23)	0.276 (0.192)	18 (16–24)	16 (15–22)	**0.001** (0.765)	0.488 (0.097)	0.071 (0.253)
Total	77 (66.3–80)	78 (70.5–82.8)	**<0.001** (0.789)	70 (68–77)	70 (67–74)	**0.004** (0.652)	0.476 (0.100)	**0.003** (0.410)
Feeding skills								
Person with dementia	2.5 (2–2.5)	2.8 (2.5–3)	**0.007** (0.475)	3 (2.5–3.5)	3.5 (3–3.5)	0.303 (0.236)	**<0.001** (0.632)	0.002 (0.439)
Caregiver	4 (4–4.5)	5.5 (5–6)	**<0.001** (0.815)	4 (4–5)	4.5 (4–5)	0.371 (0.205)	0.164 (0.195)	**<0.001** (0.539)
Environment	5 (4–6)	5.8 (5–6)	**0.012** (0.443)	6 (6–6)	6 (6–6)	0.999 (0.000)	**<0.001** (0.569)	**<0.001** (0.506)
Total	11.5 (11–12.5)	14 (13–14.5)	**<0.001** (0.842)	13.5 (13–14.5)	14 (13.5–14.5)	0.350 (0.214)	**<0.001** (.674)	0.556 (0.082)
**Knowledge test**	6 (5–6.8)	7 (6–9)	**<0.001** (0.728)	7 (5–8)	-	**-**	0.076 (0.248)	-

T0 = baseline assessment; T1 = second assessment: *p* = *p* value; *d* = Cohen’s d effect size.

The outcome measures of people with dementia showed significant differences only in meal duration at T1 (*p* = .002). Table 9 shows that oral IG intake increased, but without statistical significance as well as the EdFED score that decreased in both groups, but also without statistical significance.

**Table 9.** People with dementia’ outcomes.

	Intervention group (*n* = 19)			Control group (*n* = 16)				
	T0 Median (Q1 – Q3)	T1 Median (Q1 – Q3)	Within-group difference *p* (*d*)	T0 Median (Q1 – Q3)	T1 Median (Q1 – Q3)	Within-group difference *p* (*d*)	Between-group difference (T0) *p* (*d*)	Between-group difference (T1) *p* (*d*)
Meal length (minutes)	10.4 (9.2–15.2)	11.3 (8.6–14.2)	0.778 (0.065)	13.6 (9.7–18.2)	16.9 (12.7–19.2)	0.121 (0.388)	0.172 (0.235)	**0.002** (0.515)
Food intake	100 (81.3–100)	100 (81.3–100)	0.906 (0.027)	100 (88.5–100)	93.7 (84.9–100)	0.123 (0.386)	0.502 (0.127)	0.659 (0.085)
EdFED	6 (4.5–7.5)	5.5 (4–6)	0.077 (0.406)	6 (4.6–8.4)	5.8 (4.3–8.8)	0.964 (0.011)	0.857 (0.031)	0.271 (0.191)

T0 = baseline assessment; T1 = second assessment: *p* = *p* value; *d* = Cohen’s d effect size.

## Discussion

The purpose of this study was to analyze how FOoD-EAT, an educational intervention improved mealtime support needs of people with dementia, and affected levels of burnout, job satisfaction, and knowledge and skills among DCW. The intervention comprised three weekly sessions, focusing on theoretical knowledge and practical strategies. DCW participated in interactive training sessions to enhance their skills and confidence in assisting people with dementia with meals. The control group continued with their routine practices without any additional training or support.

The results showed that the sociodemographic characteristics of DCW and people with dementia did not differ significantly between the control and intervention groups at baseline. This suggests that both groups were comparable at the study’s outset, allowing for a fair assessment of the intervention’s effects.

At baseline, DCW identified a range of responsive behaviours and challenges encountered while assisting people with dementia during mealtimes, such as agitation, sleepiness, refusal to eat, retain food in the mouth. A notable 78.4% of DCW reported not having received prior training in mealtime support, highlighting a significant gap in their preparation. These findings align with previous studies that underline the importance of equipping DCW with skills to navigate mealtime challenges (Faraday et al., 2019; Passos et al., 2024b).

The FOoD-EAT program was well received, filling this gap by addressing both technical skills and person-centred approaches. The inclusion of “in-service” support extended classroom-based training, enabling DCW to apply their knowledge in practice and reinforcing confidence and wellbeing. The intervention had a positive impact on DCW, who in general recognized and have witnessed behaviors and situations commonly related to difficulties during mealtimes. This is evident in the reduced difficulty in assisting a person with dementia to eat and drink, as well as in the relief of physical and emotional overload. Additionally, DCW reported a greater sense of gratification from being able to assist people with dementia during mealtimes. It is important to analyze physical and emotional overload carefully, considering that mealtime assistance is just one of the responsibilities these professionals handle. Other daily tasks may also contribute to their overall workload.

Self-confidence among IG showed increases across all assessed items, with significant differences between the groups at T1. Self-confidence can be enhanced by providing knowledge, as demonstrated in previous studies, and it is critical as it likely contributes to better caregiving practices and outcomes (Elvish et al., 2018; Hughes et al., 2008; Scerri & Scerri, 2019). Additionally, self-perceived knowledge/preparation at mealtimes increased significantly in the IG, indicating that the intervention provided practical skills and knowledge that DCW could readily apply, and underscored the efficacy of the intervention in empowering DCW with the necessary tools and confidence to manage their caregiving responsibilities effectively.

The effects of the intervention were reflected in the reduction of burnout in the IG, where a significant reduction was observed in the emotional exhaustion subscale in the IG, while it increased in the CG, with a difference between the groups at T1. Both groups showed a reduction in the depersonalization subscale, but with no difference between the groups. Burnout levels for the personal accomplishment subscale showed a significant increase in the IG, indicating a tendency for individuals to negatively assess their own skills and productivity at work, potentially resulting in lower self-esteem (Bridgeman et al., 2018; Moss et al., 2016). After participating in the training sessions, participants from IG may have become more aware of job challenges, with elevated expectations, more critical self-assessment, recognition of limitations, comparisons with new standards, or difficulties adapting to changes in work practices.

It is believed that burnout is a process that starts with emotional exhaustion and progresses over time (Maslach et al., 2001). Therefore, a reduction in the emotional exhaustion subscale indicates the possibility of changes in the other components, which are the most resistant to change (Maslach et al., 2001). Providing adequate training for DCW is one of the factors that can lead to a reduction in burnout (Yeatts et al., 2018).

The intervention had a positive impact on DCW’ job satisfaction. While CG showed a decrease in all dimensions, IG participants had a significant increase in overall job satisfaction and in the intrinsic dimension. DCW who have received specialized training in providing care for individuals with dementia are more resilient to burnout and develop more job satisfaction (Coogle et al., 2006). The reduction in the extrinsic dimension in both groups could be related to the fact that job satisfaction involves other aspects that exist in the daily lives of DCW, such as overwork, dissatisfaction with salary, and relationships with coworkers and superiors, which were not covered in the context of the intervention (Acea-López et al., 2021).

Regarding feeding skills, the IG showed significant improvement in all dimensions, with even more significant results in the caregiver and environment dimensions, showing a significant difference compared to the CG. This suggests that the intervention was able to positively influence DCW to perform more actions, considered good care practices, reflecting enhanced DCW skills and confidence in managing the feeding process, and improved the feeding environment, which is an essential factor in the success of feeding activities for people with dementia. Previous studies have shown that care staff who are more skilled and who can also create a better mealtime environment are more likely to provide higher quality care, leading to better outcomes such as improved meal acceptance (Abbott et al., 2013; Chaudhury et al., 2013; Liu et al., 2019; Reed et al., 2005).

An important contribution of the intervention was related to knowledge about dementia and mealtime support needs. The IG showed a significant increase in the knowledge test, thus demonstrating the importance of the educational component offered. According to a systematic review, dementia education helps to increase DCW’ overall understanding of the dementia and challenges associated as well as their perception of competence and efficiency (Zhao et al., 2021).

Meal length significantly increased in the intervention group, indicating that people with dementia might have been receiving more attention and time during meals, which is a positive aspect of care (Charras & Frémontier, 2010; H. H. Keller et al., 2015). However, food intake and EdFED scores did not show significant changes, suggesting that other factors besides the intervention might influence these outcomes, and additional approaches may be necessary to improve food intake and nutritional status of people with dementia.

The educational intervention has provided significant benefits, particularly by enhancing the awareness and skills of DCW. This intervention has led to a better understanding of the specific needs of individuals with dementia, enabling DCW to recognize and respond to the unique challenges faced by this population. As a result, DCW were more skilled to address mealtimes challenges, facilitating a safer and more supportive dining environment for the residents. Additionally, the intervention has promoted improved planning and time management among caregivers. By equipping DCW with effective strategies, mealtime activities have become more organized and efficient, and also allowed more personalized care, respecting individual preferences and requirements of each person with dementia. This personalized care approach acknowledges and respects the dignity and autonomy of people with dementia, enhancing their overall well-being. Moreover, the intervention emphasizes the use of techniques that actively involve individuals with dementia in their mealtime routines, rather than merely replacing their participation with caregiver actions. This engagement is crucial in maintaining the cognitive and motor skills of those with dementia, as it encourages them to use their remaining abilities and promotes a sense of autonomy and purpose (H. Keller & Slaughter, 2016; Leah, 2016; Williams et al., 2020).

The findings of this study have important practical implications. Implementing specific training and intervention programs for DCW can significantly improve both the caregivers’ experience and the quality of care received by people with dementia. Educational interventions focused on enhancing confidence, reducing emotional stress, and providing practical mealtime support strategies are essential to achieve these benefits.

### Limitations and future directions

RCTs have long been considered the best study design for analyzing interventions, but they have their limitations. Most RCTs have short durations and generally small sample sizes, which often leaves them underpowered to detect differences in several effectiveness measures (Saldanha et al., 2022). There are certain limitations on this study, even with the encouraging outcomes. A small sample size and the limited number of residential care facilities enrolled may restrict the study’s capacity to be broadly applicable and to generalize results. Future studies with larger samples and in different facilities are needed to confirm these findings and explore in more detail the mechanisms through which interventions affect feeding behaviors of people with dementia and the experience of DCW. The duration of the training sessions may be another limitation of the study. Although the DCW’s time availability was limited, only 3 weekly training sessions, which totaled 6 hours, may have impacted the complete assimilation of the content. Nevertheless, DCW were supported in-service by a skilled researcher (1^st^ author) to put in practice, in real-world circumstances, what they have learned in the educational sessions.

To reduce bias, a double-blind RCT could be performed. It would be important to consider a blind evaluator to the study to apply the questionnaires and carry out the assessments, with the training sessions being conducted by one of the members of the research team. For a better understanding of the changes in burnout levels, it would be useful to conduct qualitative interviews or focus groups with care staff to explore their perceptions and experiences following the training. This could provide more detailed insights into the factors that contributed to reduced feelings of personal accomplishment and help to tailor future training programs to better support care staff. Another limitation of this study is the absence of a repeated knowledge test for the control group, which would have allowed to confirm that knowledge gains were due to the educational program alone. Future studies should consider repeated all the assessments for both groups to enhance result validity. More assessment instruments, as the Mealtime Difficulty Scale for older adults with Dementia (Jung et al., 2022) or the Eating Behavior Scale (Tully et al., 1997) can be incorporated into future studies to better understand the impact of the intervention on people with dementia.

While FOoD-EAT showed promising results in enhancing DCW’ skills and mealtime support, its integration into broader multi-component approaches could further optimize outcomes. Mealtime experiences are shaped by intrapersonal, interpersonal and environmental factors, and combining FOoD-EAT with environmental modifications could enhance both resident and caregiver experiences, aligning with evidence supporting multi-faceted interventions.

## Conclusion

The educational intervention implemented in this study suggested to be effective in improving the experience of direct care workers related to mealtime support needs of people with dementia. Participants in the intervention group showed greater knowledge and skills related to mealtime difficulties for people with dementia, in addition to more job satisfaction and lower levels of burnout. The results are particularly important as they demonstrate that targeted educational programs can effectively address specific skill gaps in DCW, ultimately leading to improved care for people with dementia. Future research should explore the long-term effects of such interventions and assess their impact on other aspects of dementia care, to build a comprehensive approach to enhancing the quality of life for people with dementia.

## Supplemental Material

Supplemental Material - Effectiveness of an educational intervention on mealtime support needs for people with dementia in residential care facilities: A cluster-randomized controlled trial Supplemental Material for Effectiveness of an educational intervention on mealtime support needs for people with dementia in residential care facilities: A cluster-randomized controlled trial by Lígia Passos, João Tavares, Melissa Batchelor, Daniela Figueiredo in Dementia
